# p66Shc activation promotes increased oxidative phosphorylation and renders CNS cells more vulnerable to amyloid beta toxicity

**DOI:** 10.1038/s41598-018-35114-y

**Published:** 2018-11-20

**Authors:** Asad Lone, Richard A. Harris, Olivia Singh, Dean H. Betts, Robert C. Cumming

**Affiliations:** 1 Department of Biology, Western University, London, Ontario N6A 5B7 Canada; 2 Department of Physiology and Pharmacology, Schulich School of Medicine and Dentistry, Western University, London, Ontario N6A 5C1 Canada

## Abstract

A key pathological feature of Alzheimer’s disease (AD) is the accumulation of the neurotoxic amyloid beta (Aβ) peptide within the brains of affected individuals. Previous studies have shown that neuronal cells selected for resistance to Aβ toxicity display a metabolic shift from mitochondrial-dependent oxidative phosphorylation (OXPHOS) to aerobic glycolysis to meet their energy needs. The Src homology/collagen (Shc) adaptor protein p66Shc is a key regulator of mitochondrial function, ROS production and aging. Moreover, increased expression and activation of p66Shc promotes a shift in the cellular metabolic state from aerobic glycolysis to OXPHOS in cancer cells. Here we evaluated the hypothesis that activation of p66Shc in CNS cells promotes both increased OXPHOS and enhanced sensitivity to Aβ toxicity. The effect of altered p66Shc expression on metabolic activity was assessed in rodent HT22 and B12 cell lines of neuronal and glial origin respectively. Overexpression of p66Shc repressed glycolytic enzyme expression and increased both mitochondrial electron transport chain activity and ROS levels in HT22 cells. The opposite effect was observed when endogenous p66Shc expression was knocked down in B12 cells. Moreover, p66Shc activation in both cell lines increased their sensitivity to Aβ toxicity. Our findings indicate that expression and activation of p66Shc renders CNS cells more sensitive to Aβ toxicity by promoting mitochondrial OXPHOS and ROS production while repressing aerobic glycolysis. Thus, p66Shc may represent a potential therapeutically relevant target for the treatment of AD.

## Introduction

Alzheimer’s disease (AD) is a chronic, neurodegenerative disorder that is characterized by a gradual development of cognitive dysfunction and memory loss. AD is currently the fourth leading cause of death in developed nations with no effective therapy currently available^1^. From a pathological perspective, AD is strongly associated with deposits of extracellular plaques and intracellular neurofibrillary tangles within broad regions of the cortex and hippocampus; events believed to be major factors contributing to disease progression^2–4^. Plaques mainly consist of the amyloid β peptide (Aβ), which arises from cleavage of the amyloid precursor protein (APP). Aβ plaque deposition begins well before the appearance of clinical symptoms of dementia^5,6^. The progressive accumulation of Aβ is strongly associated with the production of mitochondrial reactive oxygen species (ROS) and oxidative damage, leading to extensive neuronal death and synaptic loss in the AD brain^7–9^.

The brain is particularly susceptible to oxidative stress compared to other tissues due to high rates of neuronal mitochondrial metabolism and lower level of antioxidant enzyme expression^9^. Neuronal activation and increased energy metabolism are known to be intimately related. However, dysfunctional mitochondria have been observed in both neurons and astrocytes in the AD brain^10,11^. Localization of Aβ to mitochondria has been detected in both postmortem AD brain tissues as well as in transgenic mice models of AD^12^. Oligomeric forms of Aβ have been shown to interact with the mitochondrial protein Aβ binding alcohol dehydrogenase (ABAD), resulting in increased ROS production, mitochondrial impairment, and cell death^13^. Furthermore, *in vitro* studies have reported that Aβ peptides prevent nuclear encoded proteins from entering the mitochondria while activating mitochondrial fission proteins leading to decreased mitochondrial membrane potential, mitochondrial fragmentation and altered mitochondrial morphology^14,15^. ^18^F-fluoro-2-deoxy-D-glucose positron emission tomography (FDG–PET) studies have shown reduced glucose metabolism in the cortices and hippocampi of AD patients^8,16,17^. Glucose hypometabolism and reduced glucose transport have been shown to correlate with Aβ deposition in at-risk individuals of AD, as well as in patients with mild cognitive impairment^18,19^. Alterations in the relative ratio of glycolysis versus oxidative phosphorylation (OXPHOS) can significantly affect ROS production and oxidative stress in the brain^20^. Therefore, dysfunctional cerebral metabolism linked to altered mitochondrial function, glucose metabolism, and ROS production are believed to play significant roles in AD pathophysiology.

Aerobic glycolysis, also known as the Warburg effect, is defined as the preferential use of glycolysis in the presence of oxygen and is a form of metabolism frequently observed in cancer cells^21^. Interestingly, the spatial distribution of Aβ deposition correlates with elevated aerobic glycolysis in cognitively normal people^22^. It has been suggested that elevated aerobic glycolysis may arise in certain regions of the brain as a compensatory response to offset Aβ-induced ROS production^23,24^. Approximately 30% of elderly individuals accumulate significant quantities of Aβ plaques within their brains yet show no symptoms of memory loss or dementia; suggesting that cellular responses to mitigate Aβ toxicity may arise in cognitively normal individuals with high plaque deposition^25–28^. Several studies have shed light on the neuroprotective mechanisms that arise in Aβ resistant cells, including increased antioxidant enzyme expression and activity as well as reduced mitochondrial ROS production. Moreover, cells selected for Aβ resistance *in vitro* exhibit increased glucose consumption and lactate production, as well as significantly higher expression of pyruvate kinase, hexokinase, lactate dehydrogenase (LDHA), and pyruvate dehydrogenase kinase 1 (PDK1); enzymes involved in aerobic glycolysis^23,24,29,30^. Taken together, Aβ resistant cells undergo a metabolic shift away from mitochondrial dependent oxidative phosphorylation towards aerobic glycolysis to meet energy requirements. However, the upstream triggers that promote this metabolic shift, and associated resistance to Aβ toxicity, are currently unknown.

Several studies have demonstrated that the p66Shc adaptor protein is a regulator of the cellular redox state and apoptosis^31–33^. The p66Shc protein is one of three isoforms, including p46Shc and p52Shc, encoded by the *SHC1* gene. All three SHC1 isoforms contain a phosphotyrosine binding (PTB) domain, a collagen homology 1 (CH1) domain, and a Src-homology 2 (SH2) binding domain. However, due to alternative promoter usage, p66Shc contains an additional collagen homology 2 (CH2) domain^34^. All ShcA isoforms are phosphorylated at tyrosine residues in response to growth factor signaling, however p66Shc is also phosphorylated at serine 36 (S36) within the CH2 domain by kinases that are activated in response to various oxidative stressors^35–38^. As a result of S36 phosphorylation, p66Shc translocates to the mitochondria where it promotes increased ROS production, release of cytochrome-c and induction of apoptosis^38–40^. In the context of AD, recent studies have shown that Aβ exposure can promote S36 phosphorylation and activation of p66Shc in a c-jun N-terminal kinase (JNK) and mitogen-activated protein kinase kinase 6 (MKK6) dependent manner^41,42^. Aβ-induced p66Shc activation also leads to phosphorylation and repression of the Forkhead-type (FOXO) transcription factors, and a concomitant reduction in expression of antioxidant enzymes such as glutathione peroxidase-1 and catalase^43–45^. Reduced activities of these and other antioxidant enzymes have been previously reported in the AD brain as well as in transgenic mouse models of AD^46–49^. In contrast, mice with a targeted deletion of the *p66Shc* gene are phenotypically normal but live 30% longer compared to wild type mice^50^. Furthermore, p66Shc deficient cells exhibit higher expression of antioxidant enzymes and lower intracellular levels ROS levels^51–53^.

Recent evidence has also implicated p66Shc in regulating cellular metabolism. Expression and activation of p66Shc in cultured mouse embryos closely correlates with elevated mitochondrial OXPHOS and ROS production^54^. Cells lacking p66Shc exhibit lower oxygen consumption and increased lactate production, suggesting that genetic ablation of *p66Shc* leads to elevated aerobic glycolysis^55,56^. However, the relationship between p66Shc-dependent metabolic effects and cellular sensitivity to amyloid toxicity has never been examined before.

In this study, we examined the effect of p66Shc expression and activation on Aβ toxicity in CNS cells. We report that the expression and activation of p66Shc in both neuronal and glial cells increases mitochondrial electron transport chain activity while downregulating the expression of enzymes involved in glycolysis. As a consequence of elevated mitochondrial OXPHOS and ROS production, cell survival is decreased in the presence of Aβ. Our findings indicate that Aβ toxicity is strongly mediated by p66Shc-induced alterations in cellular metabolism.

## Results

### p66Shc expression and activation attenuates expression of enzymes involved in aerobic glycolysis and promotes mitochondrial OXPHOS

**Figure 1:**
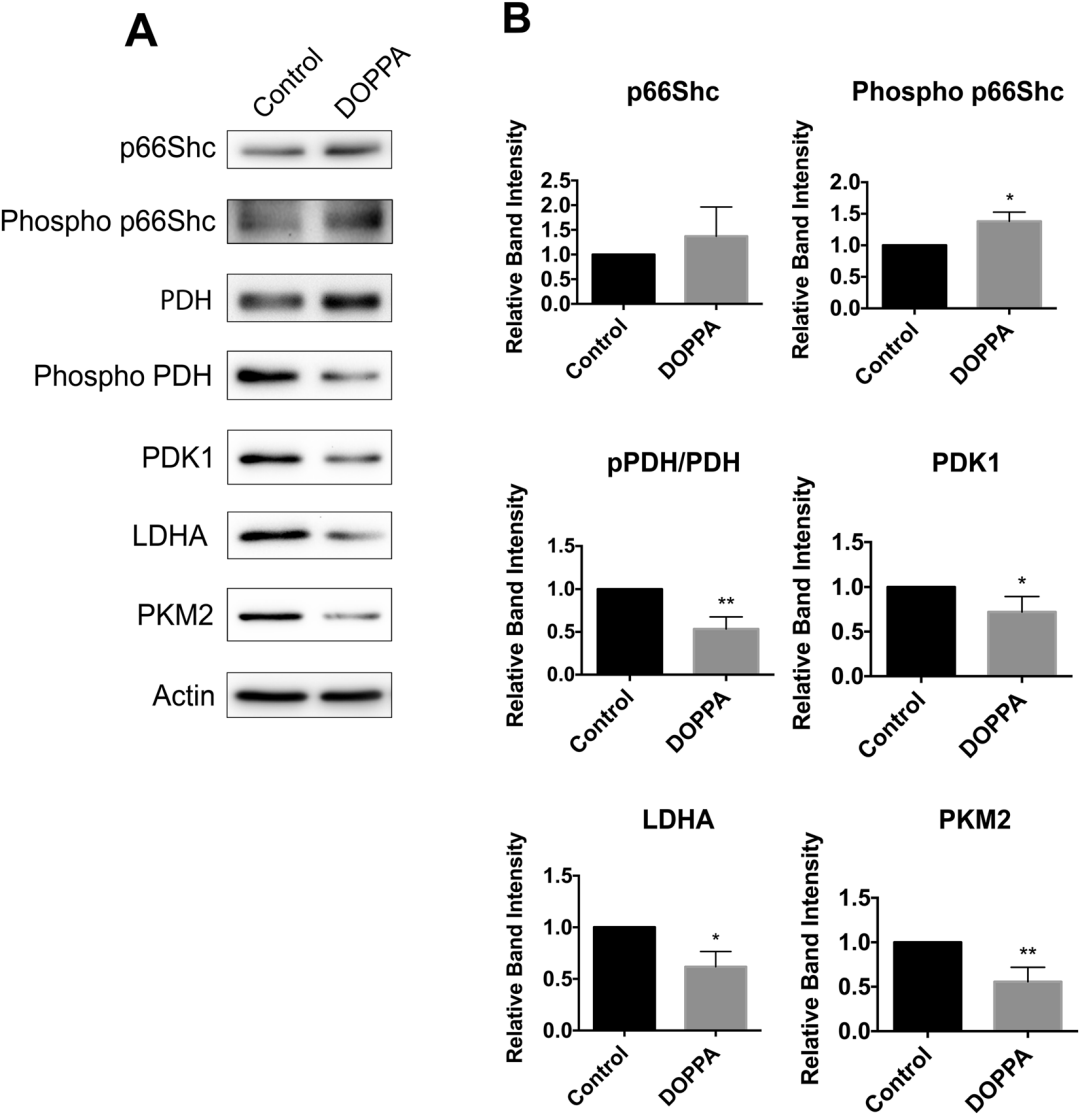
Activation of endogenous p66Shc in B12 cells promotes a reduction in the levels of aerobic glycolysis enzymes. (**A**) Immunoblot analysis of extracts from B12 cells revealed increased phosphorylation of p66Shc following 24-hour DOPPA (100 nM) exposure compared to untreated control cells. DOPPA exposure also promoted decreased phosphorylation of pyruvate dehydrogenase (PDH) and led to a reduction in levels of the aerobic glycolysis enzymes pyruvate dehydrogenase kinase 1 (PDK1), lactate dehydrogenase A (LDHA) and pyruvate kinase 2 (PKM2) compared to control cells. (**B**) Densitometric analysis of blots revealed a significant increase in S36 phosphorylation of p66Shc and a concomitant decrease in PDH phosphorylation and protein levels of PDK1, LDHA and PKM2 following DOPPA exposure. Data presented are the mean ± SEM of 3 independent experiments (*P < 0.05, **P < 0.01).

**Figure 2:**
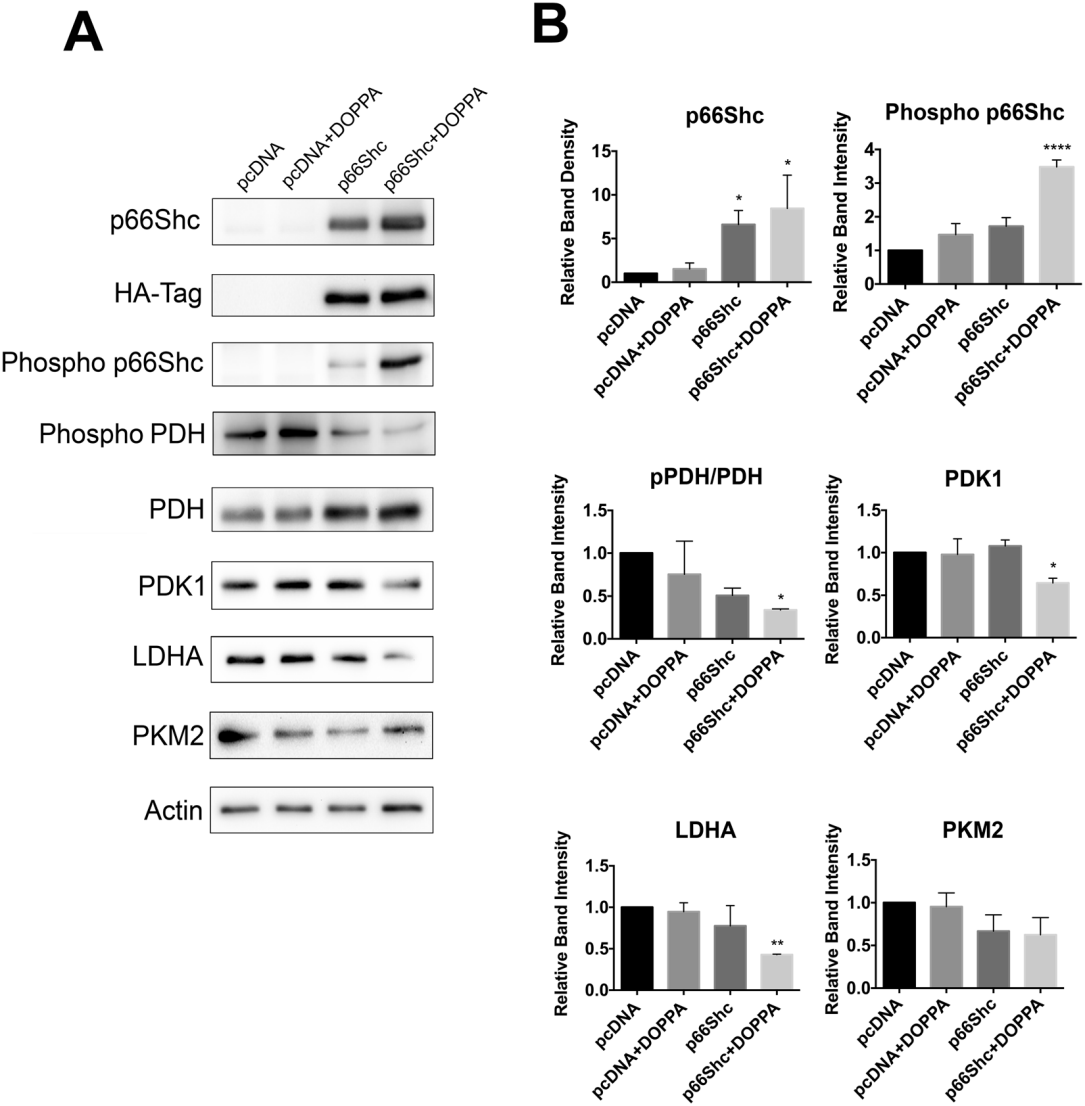
Ectopic expression and activation of p66Shc in HT22 cells promotes a reduction in aerobic glycolysis enzyme levels. (**A**) Immunoblot analysis of extracts from HT22 cells transiently transfected with either pcDNA control plasmid or a p66Shc-HA expression vector. DOPPA treatment (100 nM) promoted both increased p66Shc phosphorylation and repressed PDH phosphorylation in p66Shc-HA transfected cells. DOPPA exposure also led to a reduction in levels of the aerobic glycolysis enzymes PDK1, LDHA and PKM2 in p66Shc-HA expressing cells compared to control cells. (**B**) Densitometric analysis of blots revealed a significant increase in S36 phosphorylation of p66Shc and a concomitant decrease in PDH phosphorylation and protein levels of PDK1, LDHA and PKM2 in p66Shc-HA expressing cells following DOPPA exposure. Data presented are the mean ± SEM of 3 independent experiments (*P < 0.05, **P < 0.01).

Previous studies have demonstrated that p66Shc plays a pivotal role in mitochondrial metabolism. Restoration of p66Shc expression in p66Shc deficient HeLa cells results in elevated O_2_ consumption, while reducing the abundance of the glycolytic intermediates acetyl coenzyme A (ACoA), NADH, and lactate^55,56^. However, to our knowledge the effect of p66Shc on metabolic enzyme expression in CNS cells has not yet been examined. To this end, we investigated alterations in the expression of enzymes involved in mitochondrial OXPHOS and aerobic glycolysis following p66Shc activation in the immortalized rat glial cell line B12^57,58^, and mouse hippocampal neuronal cell line HT22^59^. We first examined endogenous p66Shc expression levels in both B12 and HT22 cells by immunoblot analysis. Endogenous p66Shc expression was detected in B12 cells (Fig. 1) but was very low in HT22 cells (Fig. 2). Protein kinase C β (PKC-β) is a kinase that phosphorylates p66Shc at the S36 residue, resulting in its activation and mitochondrial translocation^60–62^. In order to promote phosphorylation of p66Shc, cells were treated with the phorbol ester 12-Deoxyphorbol 13-phenylacetate 20-acetate (DOPPA), a specific and potent activator of PKC-β^63–66^.

**Figure 3:**
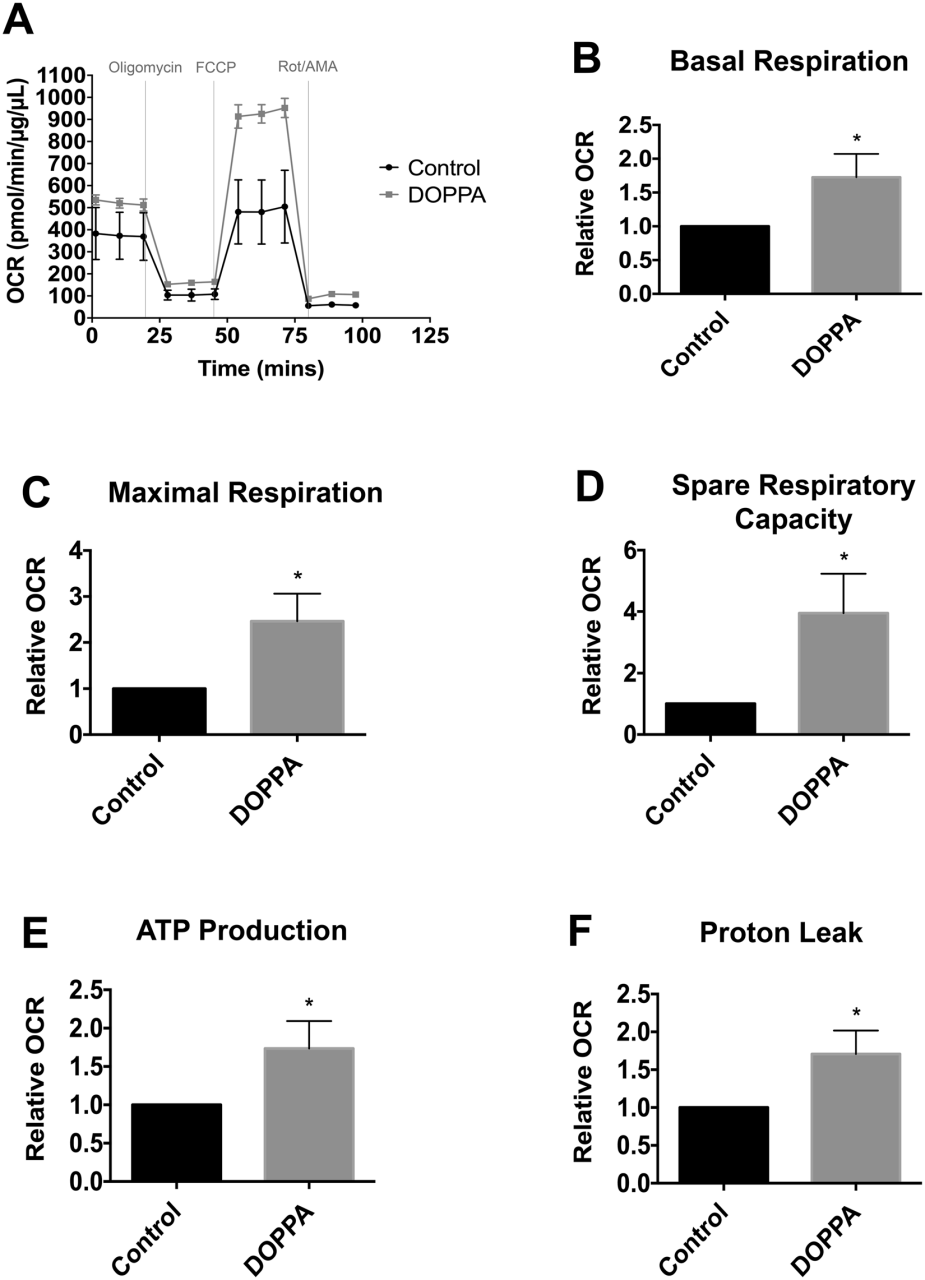
Phosphorylation and activation of endogenous p66Shc in B12 cells leads to an increase in mitochondrial oxidative metabolism. (**A**) Oxygen consumption rate of B12 cells, with and without DOPPA (100 nM) treatment for 24 hours, was measured in real-time using a Seahorse XFe24 Flux Analyzer. After normalization to protein content, B12 cells treated with DOPPA displayed significant increases in (**B**) basal respiration, (**C**) maximal respiration, (**D**) spare respiratory capacity, (**E**) ATP production, and (**F**) proton leak when compared to untreated cells. Data presented are the mean ± SEM of 3 independent experiments (*P < 0.05).

To investigate the effect of p66Shc activation on cellular metabolism, p66Shc was transiently overexpressed in HT22 cells using an HA-tagged p66Shc overexpression plasmid (hereby denoted as HT22^p66Shc^). DOPPA-induced phosphorylation of p66Shc was observed in both B12 and HT22^p66Shc^ cells (Figs 1 and 2). We then looked at the resulting effect of p66Shc activation on the expression of proteins involved in OXPHOS and aerobic glycolysis by immunoblot analysis. Pyruvate dehydrogenase kinase 1 (PDK1) is involved in the phosphorylation and inhibition of pyruvate dehydrogenase (PDH), an enzyme which converts pyruvate to ACoA for entry into the TCA cycle. Lactate dehydrogenase A (LDHA) is responsible for converting pyruvate to lactate, a metabolite widely used as a marker for glycolysis. Pyruvate kinase 2 (PKM2) is an alternatively spliced isoform of pyruvate kinase that favours glycolysis and lactate production^67,68^. A significant decline in levels of the glycolytic enzymes PDK1 and LDHA in B12 and HT22^p66Shc^ cells was observed following DOPPA-induced phosphorylation of p66Shc (Figs 1 and 2). PKM2 levels also showed a significant reduction in B12 cells, whereas a modest but non-significant decrease of PKM2 was observed in HT22^p66Shc^ cells (Figs 1 and 2). In addition, a significant decrease in phosphorylated PDH was detected in both cell lines expressing active p66Shc (Figs 1 and 2). HT22 cells transfected with an empty pcDNA vector and treated with DOPPA showed no change in either OXPHOS or glycolytic enzyme expression (Figs 1 and 2). To validate these findings, we also measured the oxygen consumption rate (OCR) in real time, as a measure of OXPHOS, in live B12 cells with or without p66Shc activation using the Seahorse XFe24 flux analyzer (Fig. 3A). A significant increase in the rates of basal as well as maximal respiration were observed in B12 cells following p66Shc activation via DOPPA treatment (Fig. 3B,C). Furthermore, B12 cells with phosphorylated and active p66Shc also had significantly higher spare respiratory capacity, ATP production, and Proton Leak when compared to control cells (Fig. 3D–F). Taken together, p66Shc expression and activation promotes alterations in metabolic enzyme expression which favour OXPHOS while suppressing glycolysis.

### Expression and activation of p66Shc promotes increased mitochondrial electron transport chain activity and ROS production

**Figure 4:**
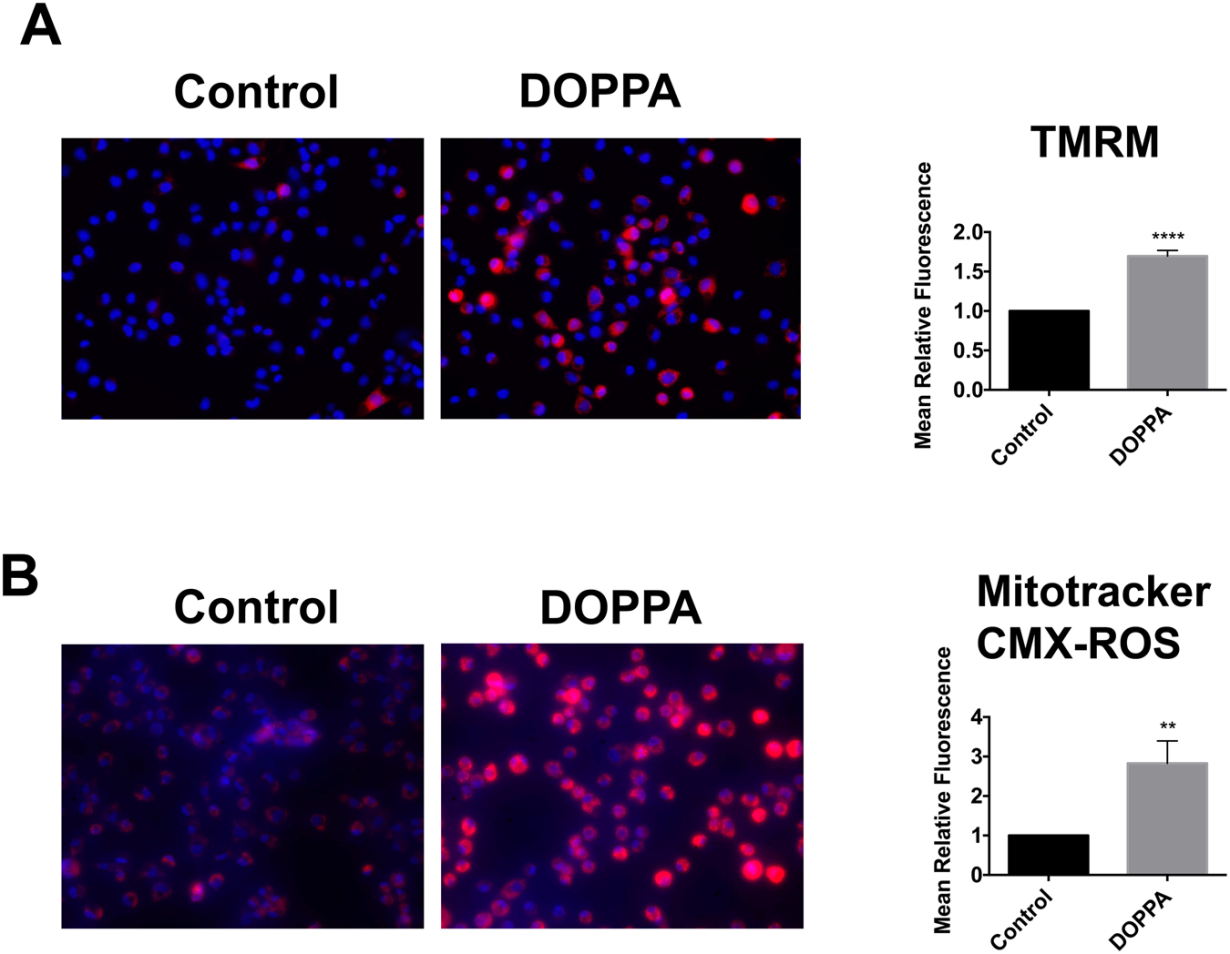
p66Shc activation promotes an increase in mitochondrial membrane potential (∆𝜓m) and ROS production in B12 cells. (**A**) B12 cells were stained with the ∆𝜓m sensitive fluorochrome TMRM (red), while nuclei were stained with Hoechst stain (blue) and visualized by fluorescence microscopy. Quantification of TMRM fluorescence (right panel) revealed a significant elevation of ∆𝜓m in DOPPA (100 nM) treated B12 cells when compared to untreated control cells. **(B)** B12 cells were stained with Mitotracker CMX-ROS (Red) and visualized by fluorescence microscopy. Quantification of Mitotracker CMX-ROS (right panel) revealed a significant increase in mitochondrial ROS production following DOPPA treatment (100 nM) compared to control cells. Data presented are the mean ± SEM of 3 independent experiments (**P < 0.01; ****P < 0.001).

**Figure 5:**
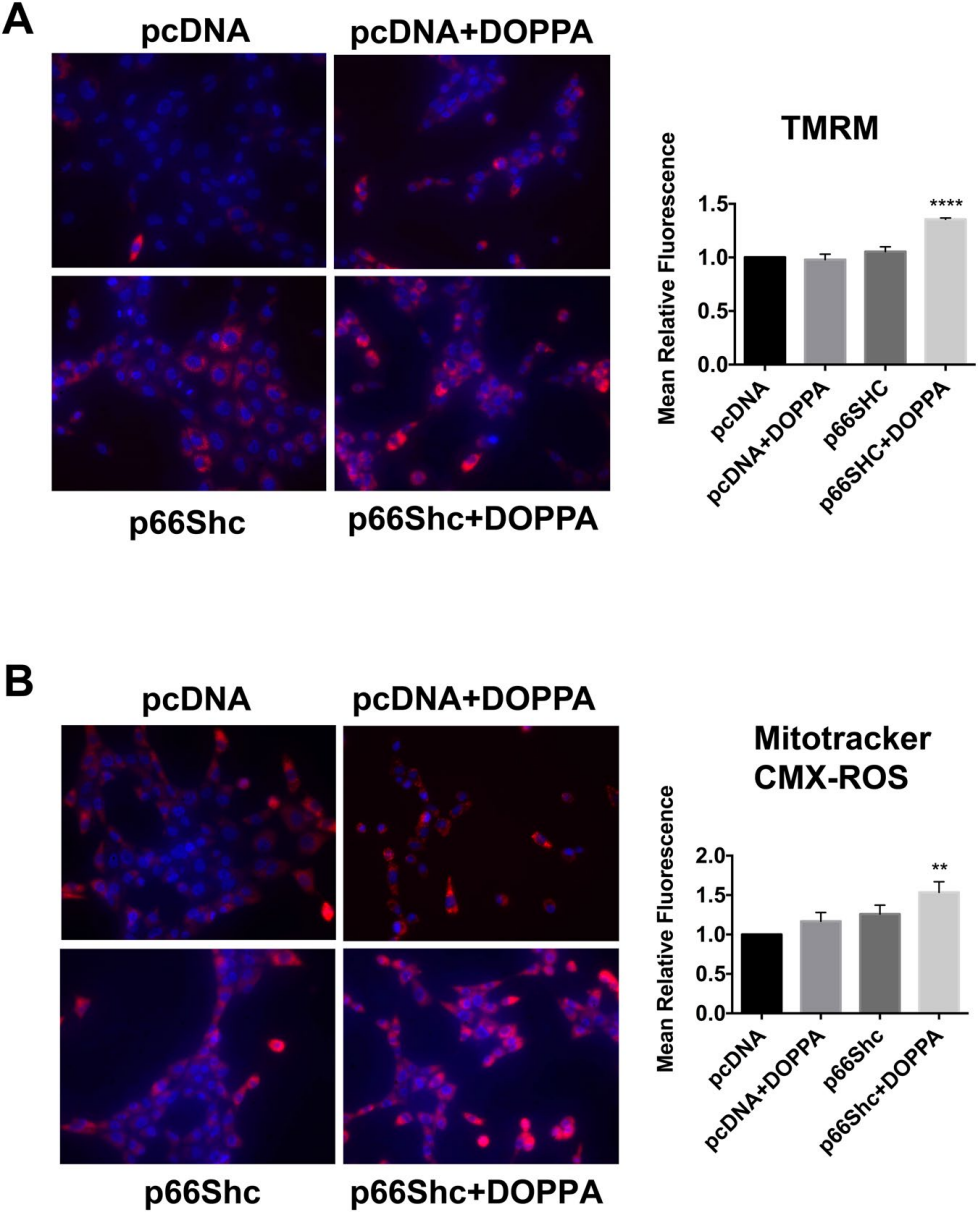
Ectopic expression of p66Shc in HT22 cells promotes increased mitochondrial membrane potential and ROS production following DOPPA exposure. (**A**) HT22 cells were transfected with either pcDNA or a p66Shc-HA expression plasmid, treated with DOPPA (100 nM) and stained with TMRM. Stained cells were visualized by fluorescence microscopy and fluorescence intensity was quantified (right panel). (**B**) HT22 cells transfected as indicated and treated with DOPPA (100 nM) were stained with Mitotracker CMX-ROS and visualized by fluorescence microscopy. Fluorescence intensity of stained cells was quantified (right panel). HT22 cells transfected with p66Shc and treated with DOPPA exhibited significantly higher TMRM and Mitotracker CMX-ROS staining compared to pcDNA control transfected cells. Nuclei were stained with Hoechst stain (blue). Data presented are the mean ± SEM of 3 independent experiments (**P < 0.01; ****P < 0.001).

Maintenance of mitochondrial membrane potential (∆𝜓m) is essential for ATP production and cell viability. The fluorochrome tetramethylrhodamine methyl ester (TMRM) is frequently used to measure ∆𝜓m, and corresponding changes in ETC and OXPHOS activity^69–72^. In addition, elevated ETC and OXPHOS are also frequently associated with increased mitochondrial ROS production, which is detectable with the fluorochrome Mitotracker CMXRos^73^. We therefore evaluated the effect of p66Shc activation on both ∆𝜓m and mitochondrial ROS levels. B12 cells treated with DOPPA exhibited a significant increase in ∆𝜓m compared to control treated cells (Fig. 4A). As expected, a significant increase in mitochondrial ROS production was also observed in DOPPA-treated B12 cells (Fig. 4B). Similarly, HT22^p66Shc^ cells treated with DOPPA also exhibited a significant increase in both ∆𝜓m and ROS production compared to DOPPA treated cells transfected with pcDNA (Fig. 5). Overall, these findings demonstrate that the expression and activation of p66Shc enhances mitochondrial metabolism by increasing ETC activity, and consequently ROS production.

### p66Shc silencing shifts cellular metabolism towards aerobic glycolysis

**Figure 6:**
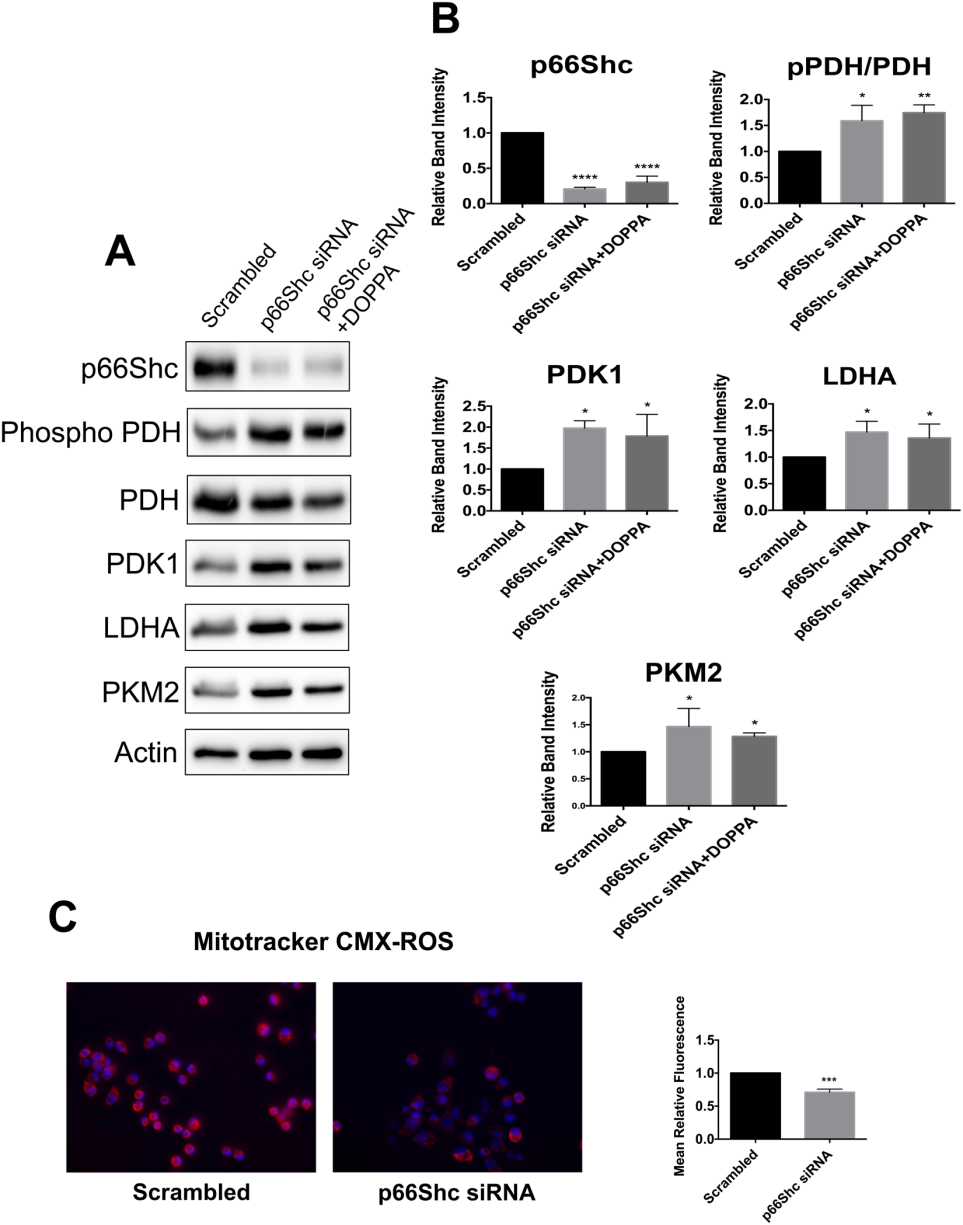
Silencing *p66Shc* expression promotes aerobic glycolysis while reducing mitochondrial ROS production. (**A**) Immunoblot analysis of extracts from B12 cells transfected with p66Shc specific siRNA. Knockdown of *p66Shc* expression resulted in elevated levels of PDK1, LDHA and PKM2 in addition to increased phosphorylation of PDH. This effect was also observed in B12 cells with silenced *p66Shc* expression treated with DOPPA. (**B**) Densitometric analysis of immunoblots. (**C**) Mitotracker CMX-ROS (red) staining was significantly decreased in B12 cells with silenced p66Shc expression when compared to control cells. Nuclei were stained with Hoechst stain (blue). Data presented are the mean ± SEM of 3 independent experiments (*P < 0.05, **P < 0.01; ***P < 0.001).

Cells in which *p66Shc* transcript abundance is knocked down exhibit increased glucose uptake, elevated production of glycolytic intermediates, as well as diminished O_2_ consumption; all hallmarks of the Warburg effect^55,56^. Hence, we silenced endogenous *p66Shc* expression in B12 cells using *p66Shc* specific siRNAs. After confirming *p66Shc* knockdown (Fig. 6A,B), we analyzed the expression of key enzymes involved in OXPHOS and aerobic glycolysis by immunoblot analysis. A significant increase in expression of the glycolytic enzymes PDK1, LDHA, and PKM2 was detected following knockdown of *p66Shc* (Fig. 6A,B). In addition, increased phosphorylation of PDH, was also observed following *p66Shc* knockdown (Fig. 6A,B). B12 cells transfected with *p66Shc* siRNA and also treated with DOPPA exhibited the same alterations in metabolic enzyme levels as cells transfected with *p66Shc* siRNA alone; demonstrating that DOPPA mediated effects on metabolism occur in a p66Shc-dependent manner (Fig. 6A,B). Since aerobic glycolysis does not use oxygen to produce ATP, a shift away from mitochondrial metabolism is associated with reduced ROS production. We therefore examined ROS levels in cells with reduced *p66Shc* expression using the fluorescent dye Mitotracker CMXRos. B12 cells transfected with *p66Shc* specific siRNAs had significantly lower ROS production when compared to cells transfected with scrambled siRNAs (Fig. 6C). Thus, silencing *p66Shc* expression promotes aerobic glycolysis while reducing mitochondrial ROS production.

### Aβ phosphorylates p66Shc and promotes OXPHOS while repressing aerobic glycolysis

**Figure 7:**
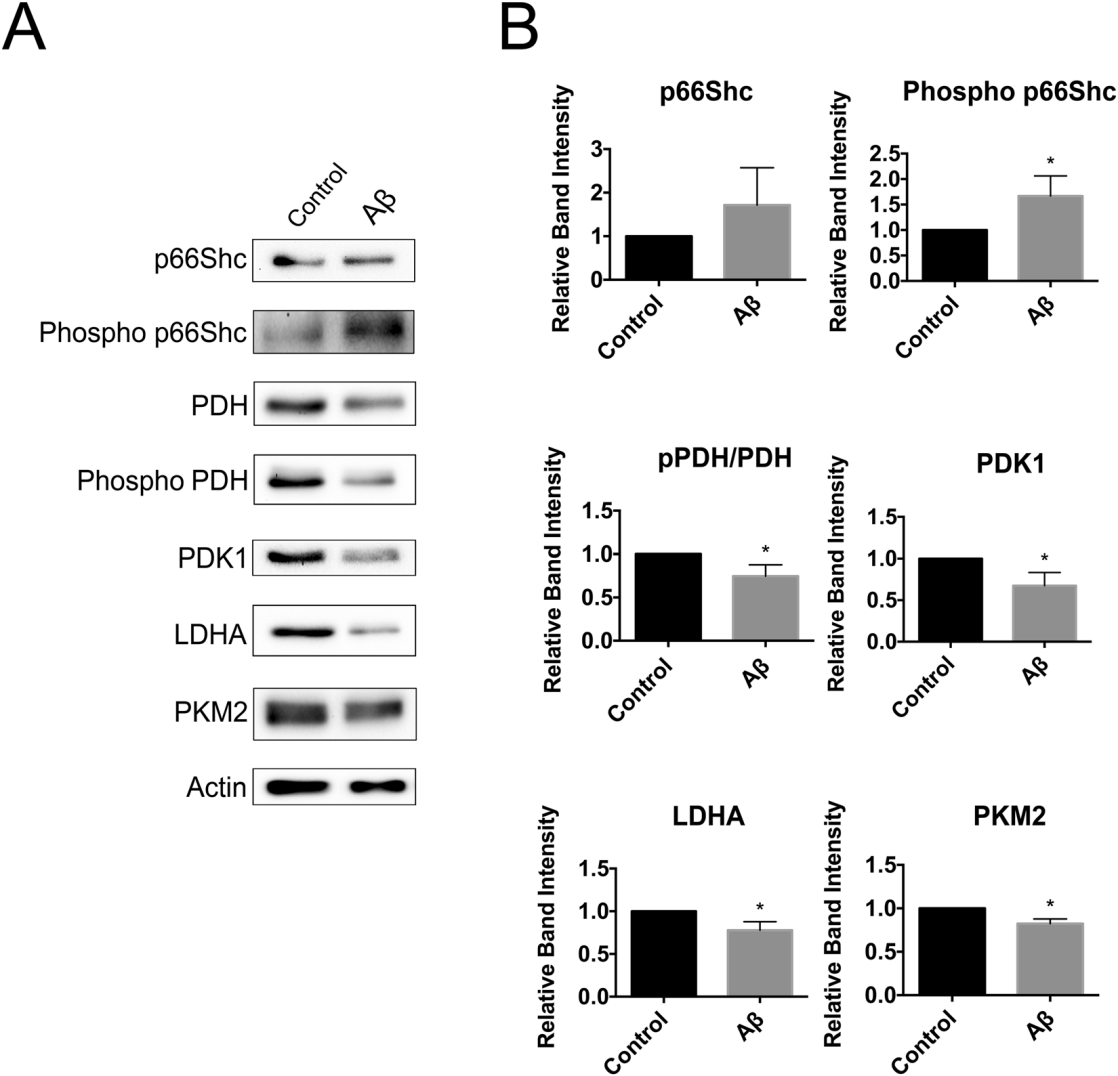
Aβ exposure promotes p66Shc activation and a reduction in aerobic glycolysis enzyme levels in B12 cells. (**A**) Immunoblot analysis of B12 cells treated with Aβ_1–42_ (20 µM) for 24 hours. (**B**) Densitometric analysis of immunoblots revealed a significant increase in p66Shc phosphorylation and a concomitant decrease in PDH phosphorylation and levels of PDK1, LDHA, and PKM2 following Aβ exposure. Data presented are the mean ± SEM of 3 independent experiments (*P < 0.05).

**Figure 8:**
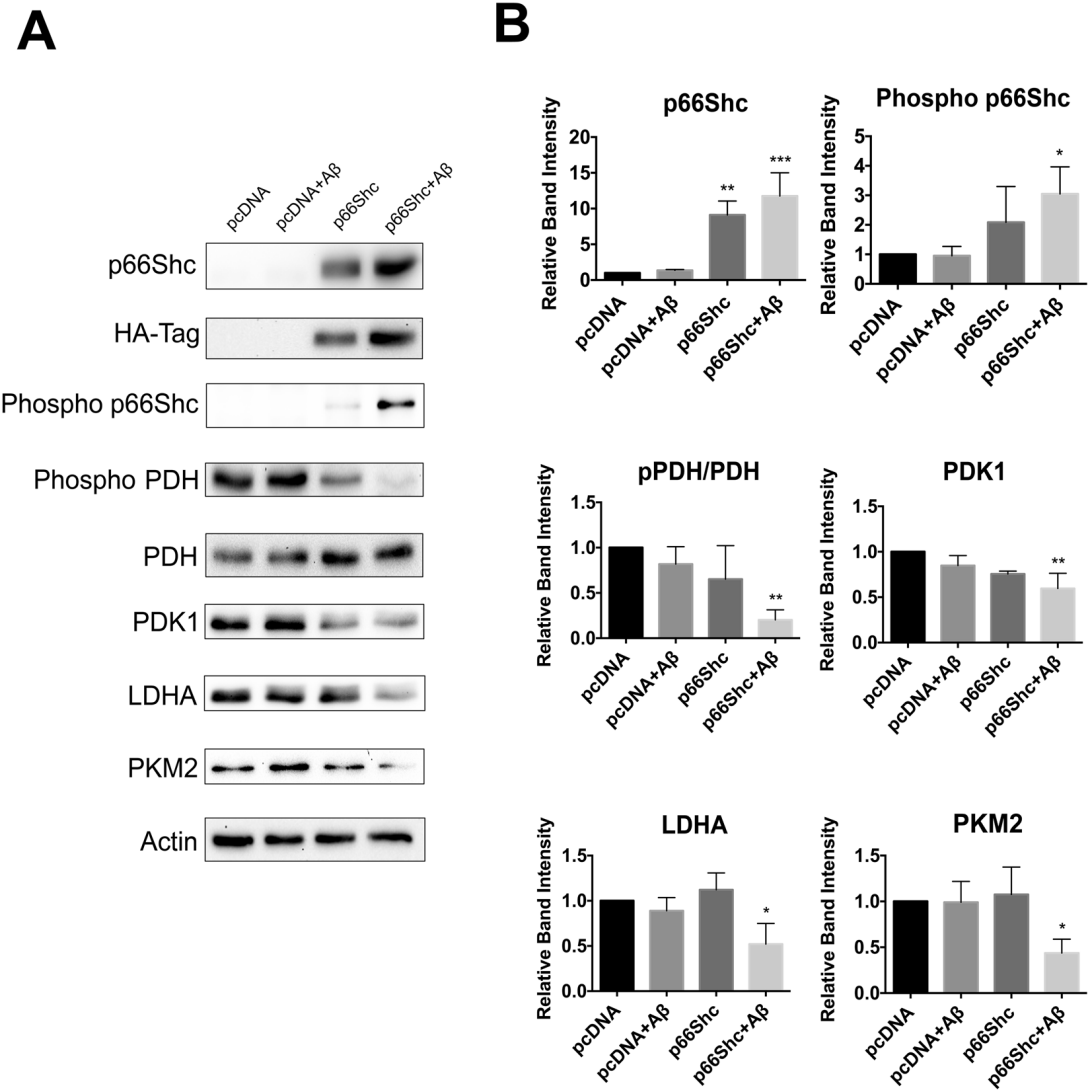
Aβ exposure promotes activation of ectopically expressed p66Shc in HT22 cells and a reduction in aerobic glycolysis. (**A**) Immunoblot analysis of extracts from HT22 cells transfected with the indicated plasmids and treated with Aβ_1–42_ (20 µM) for 24 hours. (**B**) Densitometric analysis of immunoblots revealed that Aβ exposure promoted a significant increase in p66Shc phosphorylation while repressing PDH phosphorylation. Aβ treatment also promoted a significant decrease in the levels of PDK1, LDHA, and PKM2 in HT22 cells ectopically expressing p66Shc compared to pcDNA transfected control cells. Data presented are the mean ± SEM of 3 independent experiments (*P < 0.05; **P < 0.01; ***P < 0.001).

Previous studies have demonstrated that Aβ exposure elicits increased phosphorylation of p66Shc at S36^41,42^. To determine if Aβ-mediated effects on metabolism correlate with the activation state of p66Shc, we treated B12 and HT22^p66Shc^ cells with Aβ_1–42_ and examined OXPHOS and aerobic glycolysis enzyme expression by immunoblot analysis. B12 cells treated with Aβ_1–42_ exhibited increased p66Shc phosphorylation (Fig. 7). As anticipated, a significant reduction was observed in the phosphorylation state of PDH, and expression of the glycolytic enzymes PDK1, LDHA, and PKM2 (Fig. 7). HT22^p66Shc^ cells treated with Aβ_1–42_ also exhibited a significant increase in p66Shc phosphorylation, leading to a reduction in phospho-PDH, PDK1, LDHA, and PKM2 levels (Fig. 8). HT22 cells transfected with an empty pcDNA vector and treated with Aβ_1–42_ showed no expression changes in either OXPHOS or glycolytic enzymes (Fig. 8). Thus, Aβ exposure promotes changes in expression of metabolic enzymes favouring OXPHOS in a manner that closely parallels p66Shc activation.

### Expression and activation of p66Shc increases sensitivity to Aβ toxicity

**Figure 9:**
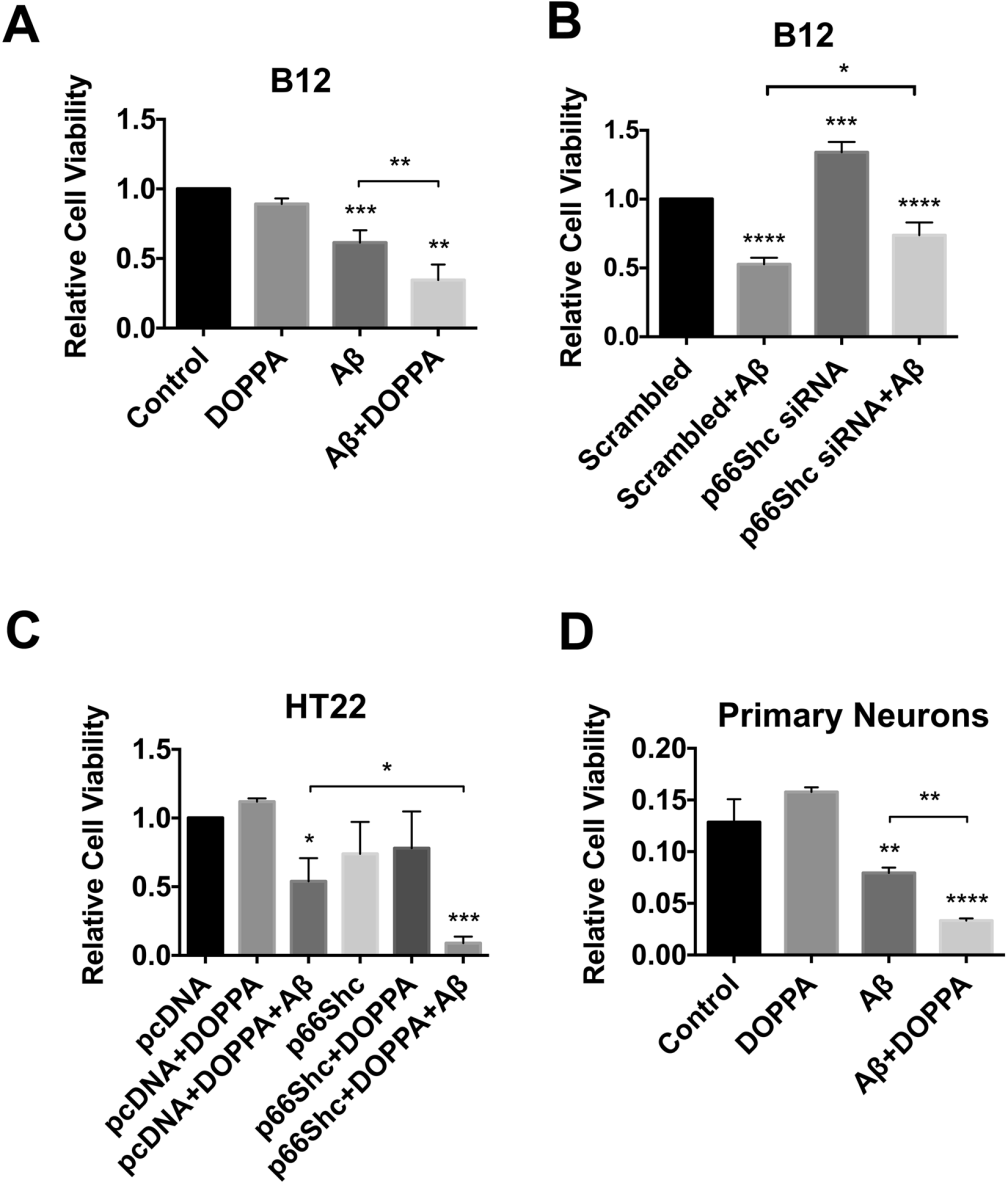
p66Shc activation enhances Aβ toxicity. (**A**) Treatment of B12 cells with both Aβ_1–42_ (20 µM) and DOPPA (100 nM) was significantly more toxic than Aβ treatment alone. (**B**) Silencing of p66Shc expression in B12 cells led to reduced Aβ-induced toxicity compared to B12 cells transfected with control siRNA and treated with Aβ. (**C**) HT22 cells ectopically expressing p66Shc and treated with DOPPA (100 nM) exhibited significantly decreased viability following Aβ treatment compared to pcDNA control cells treated with both agents. (**D**) DOPPA induced activation of p66Shc exacerbated Aβ toxicity in mouse primary cortical neurons. Data presented are the mean ± SEM of 3 independent experiments (*P < 0.05; **P < 0.01; ***P < 0.001; ****P < 0.0001).

Various forms of oxidative stressors have been shown to be more damaging to cells and tissues expressing p66Shc^41,50,52,53,74,75^. However, most studies examining p66Shc expression and oxidative stress-induced toxicity have not distinguished between the non-phosphorylated and phosphorylated forms of p66Shc. We have shown that phosphorylated p66Shc promotes OXPHOS and increased ROS production. To address the question of whether the active form of p66Shc enhances Aβ toxicity by elevating OXPHOS and mitochondrial ROS production, we exposed B12 and HT22^p66Shc^ cells to Aβ_1–42_ with or without DOPPA treatment, and quantified cell survival using the MTT assay. DOPPA treatment alone had no effect on cell viability in B12 cells (Fig. 9A). However, DOPPA exposure significantly enhanced Aβ_1–42_ toxicity in B12 cells. In contrast, transient knock down of endogenous p66Shc expression in B12 cells using p66Shc specific siRNAs, resulted in significantly higher cell survival after Aβ_1–42_ treatment when compared cells transfected with scrambled siRNA (Fig. 9B). Concurrent Aβ_1–42_ and DOPPA treatment of HT22^p66Shc^ cells resulted in significantly decreased cell viability compared to cells transfected with a control vector and treated with Aβ_1–42_ alone (Fig. 9C). To further confirm these findings, we investigated whether active p66Shc increased sensitivity to Aβ toxicity using mouse cortical neuronal cultures. Primary neurons with or without DOPPA treatment were exposed to Aβ_1–42_ at 6 days *in vitro* (DIV). As expected, primary neurons with active p66Shc showed a significant reduction in cell survival when compared neurons that lacked active p66Shc and treated with Aβ_1–42_ (Fig. 9D). Collectively, these data demonstrate that p66Shc activation potentiates Aβ_1–42_ toxicity.

Aβ exposure has previously been shown to trigger apoptosis in a JNK dependent manner^41,76^. Moreover, Aβ-induced JNK activation also promotes increased phosphorylation of p66Shc^41^. To determine if Aβ-induced activation of p66Shc was mediated by JNK in B12 and HT22^p66Shc^ cells, we examined the phosphorylation status of JNK following Aβ exposure. Although Aβ exposure significantly induced p66Shc phosphorylation in B12 cells, there was no effect on JNK phosphorylation (Figure S1). In addition, Aβ-induced p66Shc phosphorylation in HT22^p66Shc^ cells also occurred in the absence of any increase in JNK phosphorylation (Figure S2). These findings indicate that Aβ-induced activation of p66Shc is not mediated by the JNK pathway in the cell models employed in this study.

## Discussion

In this study we demonstrate that the expression and activation of p66Shc in CNS cells significantly increases OXPHOS, while downregulating aerobic glycolysis. Specifically, we observed a significant decline in levels of the glycolytic enzymes PDK1, LDHA, and PKM2 in cells expressing activated p66Shc. We also observed significantly reduced phosphorylation of PDH following p66Shc activation. Reduced PDH phosphorylation promotes increased activity of the PDH complex and enhanced flux of glycolytic intermediates into the TCA cycle for energy production^77–79^. Two previous *in vitro* studies, using mouse embryonic fibroblasts (MEFs) and human HeLa cells, demonstrated that the expression of p66Shc increased O_2_ consumption while reducing the production of glycolytic intermediates^55,56^. The findings presented here provide further support that p66Shc acts as an upstream inhibitor of aerobic glycolysis while at the same time promoting increased OXPHOS in cells of glial and neuronal origin. The mechanism by which p66Shc modulates metabolism is poorly understood but RNA sequencing analysis of wild type and p66Shc knock out MEFs, revealed no differences in transcript abundance for genes encoding glycolytic enzymes; suggesting that p66Shc likely regulates metabolism through signaling and/or post-translational processes^56^. Post-translational modifications of diverse metabolic enzymes regulate activation of aerobic glycolysis and reprograming of cell metabolism in cancer^80^. Thus, future studies examining the effect of p66Shc activation on post-translational modifications of aerobic glycolysis enzymes are warranted.

Increased ROS production is associated with age- and disease-dependent loss of neurons leading to cognitive dysfunction^9–12^. Although Aβ accumulation has historically been perceived as a critical driver of AD pathogenesis, the failure of clinical trials targeting Aβ have challenged this theory^81,82^. Moreover, several lines of evidence suggest that mitochondrial-derived ROS enhances amyloid precursor protein processing and Aβ production^83,84^. It is therefore possible that age-dependent changes in metabolism and mitochondrial dysfunction, possibly mediated by p66Shc activation, are key initiating events that trigger Aβ production resulting in a feed forward mechanism to further enhance p66Shc activation and mitochondrial ROS levels in a vicious cycle. Thus, Aβ accumulation may not necessarily initiate neurodegenerative processes in AD but rather may potentiate p66Shc activation and age-related mitochondrial impairment. The question arises as to what signaling pathways are perturbed with age that subsequently trigger elevated mitochondrial ROS production and possibly increase Aβ levels?

Neuroinflammatory changes, including microglial activation and production of inflammatory cytokines, are frequently detected in neurodegenerative diseases and normal aging^85^. The mitogen-activated protein kinase (MAPK) pathway is significantly activated during neuroinflammation and in response to oxidative stress^86^. MAPKs, including JNK and extracellular signal-regulated kinase (ERK), in addition to protein kinases such Src and PKC, are responsible for p66Shc phosphorylation; depending on the cellular context and nature of the stimulus^87^. Although JNK activation is implicated in Aβ-induced neuronal death both *in vitro* and *in vivo*^76,88,89^, we did not observe increased JNK phosphorylation following Aβ exposure in both B12 and HT22^p66Shc^ cells, indicating that Aβ exposure possibly activates other kinases that phosphorylate p66Shc. ERKs have been reported to be significantly upregulated in cell culture and animal models of AD, and higher ERK activation has been detected in AD brain extracts when compared to control subjects^85,90,91^. Inhibition of ERKs and other kinases that phosphorylate p66Shc, including PKC-β, have been shown to reduce oxidative stress and increase cellular resistance to various stressors^85,90^. In a recent study, pharmacological inhibition of PKC-β *in vitro* prevented S36 phosphorylation of p66Shc and lowered ROS during hyperglycemic stress^92^. Therefore, targeting upstream activators of p66Shc, such as ERK and PKC-β, may be an effective strategy to attenuate Aβ toxicity.

In addition to promoting increased ROS production, activation of p66Shc also leads to downregulation of anti-oxidant enzyme expression both *in vitro* and *in vivo*. Aged mice exhibit an increase in S36 phosphorylation of p66Shc and lower levels of catalase, superoxide dismutase 1 (SOD1) and glutathione peroxidase 1 (GPX1) compared to newborns^93^. Previous studies have identified p66Shc as a negative regulator of the Forkhead (FOXO) transcription factors, a family of proteins which when activated lead to the transcriptional activation of a host of ROS scavenging enzymes e.g. catalase and manganese superoxide dismutase (MnSOD). *In vitro* studies have shown that treatment of cells with H_2_O_2_ or Aβ results in increased phosphorylation and activation of p66Shc and subsequent inhibition of FOXO3a resulting in downregulation of the downstream transcriptional targets catalase and MnSOD^41,94^. Thus, p66Shc activation exacerbates Aβ toxicity by promoting increased mitochondrial ROS production while at the same time repressing antioxidant enzyme expression. Interestingly, cells selected for resistance to Aβ toxicity exhibit both an increase in both glycolytic and antioxidant enzyme expression; proteins repressed by activated p66Shc^23,24,29^.

In this study, we showed that activation of p66Shc potentiates Aβ toxicity in both B12 and HT22 cells; an event closely linked to repressed aerobic glycolysis. Interestingly, CNS cells selected for resistance to Aβ toxicity, or cells overexpressing either LDHA or PDK1, exhibit a metabolic switch from OXPHOS to aerobic glycolysis^23,24^. As a result of this metabolic reprogramming, Aβ-resistant cells restrict the amount of glycolytic flux through the mitochondria, leading to lowered mitochondrial membrane potential and ROS production. In contrast, chemically or genetically inhibiting LDHA or PDK1 re-sensitizes resistant cells to Aβ toxicity, indicating that aerobic glycolysis plays a key role in modulating CNS cell sensitivity to toxins^23^. The role of p66Shc in modulating brain metabolism and cellular sensitivity to Aβ *in vivo* is currently unknown.

It is well established that advanced age is one of the strongest risk factors for AD. The likelihood of developing AD doubles about every five years after age 65 and reaches nearly 50 percent by the age of 85^95^. Thus, molecular mechanisms underlying the aging process likely predispose the brain to the toxic effects of pathogenic proteins involved in AD. Interestingly, brain aerobic glycolysis declines naturally with age^96^. In contrast, the distribution of aerobic glycolysis in the brain correlates spatially with Aβ deposition in cognitively normal individuals and in AD patients^22^. Elevated glycolysis has also been detected in the brains of individuals with mild cognitive impairment^97^. Moreover, patients with elevated glycolysis and low amyloid levels did not convert to AD over the course of 18 months, whereas amyloid-positive individuals with low glycolysis did convert to AD within the same time period^97^. These findings raise the possibility that elevated aerobic glycolysis occurs as a compensatory protective mechanism to counter Aβ toxicity in the brains of individuals with high Aβ deposition but with little to no cognitive impairment. Genes that regulate age-dependent alterations in cerebral metabolism and toxin sensitivity are currently unknown but *p66Shc* represents a strong candidate for future evaluation.

In this study, we demonstrated that knockdown of endogenous *p66Shc* significantly upregulated key glycolytic enzymes, such as LDHA, the enzyme that catalyzes the conversion of pyruvate to lactate. Indeed, previous studies have illustrated that silencing of *p66Shc* expression not only promoted aerobic glycolysis, but also increased lactate production^55,56^. Interestingly, not only can the brain can access circulating lactate for energy production, but it can use this metabolite as a preferential fuel over glucose. Earlier work has shown that when lactate is used as a primary fuel, glucose utilization decreases in cultured neurons and astrocytes, and also in the whole brain^98–101^. The enzymatic conversion of lactate to pyruvate for utilization in the TCA cycle is thermodynamically favorable when compared to the conversion of glucose to pyruvate, as the latter reaction requires ATP consumption, however the former does not^102^. Hence, lactate use as a fuel is glucose sparing, which may be a beneficial metabolic strategy in the aged brain to compensate for age related reductions in glucose metabolism. Moreover, lactate is required for long term memory formation, and emerging evidence has also identified lactate as a signaling molecule in the brain promoting gene expression linked to synaptic plasticity^103–106^. Lactate has also been shown to be neuroprotective, especially in the face of glutamate induced excitotoxicity^107,108^. There is evidence of increased p66Shc levels in the hippocampus and frontal cortex of AD patient brain extracts^91^. Thus, therapeutic strategies which either target p66Shc or increase lactate production may prevent cognitive decline associated with aging or AD.

In the context of aging, adult wild type mice exhibit a significant increase in both p66Shc protein levels and S36 phosphorylation in multiple tissues when compared to newborns; events associated with higher mitochondrial H_2_O_2_ production^93^. A significant increase in p66Shc mRNA levels were also found in the brains of aged rats relative to young animals^109^. Deletion of the p66Shc gene in mice results in increased resistance to stress and an approximate 30% extension of life span. These mice show an apparently normal phenotype and are characterized by a decreased incidence of aging-associated diseases^50,110^. From a metabolic perspective, p66Shc knockout mice exhibit increased insulin sensitivity and glucose tolerance, and are more resistant to weight gain when fed a high fat diet^111,112^. Thus, the age-related increase in p66Shc expression and activation may alter the metabolic state of the brain and render neurons more susceptible to Aβ induced toxicity.

Interestingly, deletion of the *p66Shc* gene in mice leads to an improvement in age-dependent cognitive decline, as well as significant increases in levels of the neurotrophin brain derived neurotrophic factor (BDNF) in the hippocampus and sustained hippocampal neurogenesis^113,114^. Moreover, genetic ablation of *p66Shc* in an AD mouse model (APP/PS1) leads to a reversal of age-dependent cognitive decline, independent of Aβ levels and plaque formation. The improvement in cognitive function in APP/PS1 mice lacking *p66Shc* was associated with a reversal of mitochondrial complex I dysfunction, increased ATP production, and reduced ROS levels in cortical tissue^115^. A recent study also revealed that *p66Shc* knockout (−/−) mice were protected from diabetes-induced cognitive decline, which correlated with a decrease in oxidative stress and pro-inflammatory markers in the brain^116^. Interestingly, diabetes induction promoted an increase in microglia, a pro-inflammatory cell type, in wild-type but not in p66Shc^−/−^ mice^116^. Numerous studies have shown that chronic microglial inflammatory activity is a major contributing factor to AD pathogenesis^117^.

In conclusion, our work demonstrates that expression and activation of p66Shc in CNS cells promotes mitochondrial metabolism while suppressing glycolytic enzyme expression. As a consequence, ETC activity and ROS production is elevated. CNS cells that express activated p66Shc are therefore more sensitive to Aβ induced toxicity. However, silencing p66Shc expression shifts the metabolic state of a cell away from OXPHOS and towards aerobic glycolysis, thereby lowering ROS levels and promoting stress resistance against Aβ. Our findings suggest that agents which either target p66Shc, including upstream activating kinases, or drugs which enhance aerobic glycolysis may have therapeutic relevance for the treatment of AD and possibly other age-dependent neurodegenerative disorders.

## Materials and Methods

### Cell Culture

HT-22 and B12 immortalized cell lines were a gift from Dr. Dave Schubert (Salk Institute for Biological Sciences, California, US). Both cell lines were cultured in DMEM (Lonza) supplemented with 10% FBS (Corning) and 1% penicillin and streptomycin (pen/strep) (Gibco). Cell transfections were performed using Opti-MEM (Gibco) and Lipofectamine 2000 (Invitrogen) according to manufacturer’s instructions, either using 3 µg DNA and 5 µL Lipofectamine 2000 in a 60 mm cell culture dish, or 1.2 µg DNA and 2 µL Lipofectamine 2000 in a 35 mm cell culture dish. Transfection media was replaced with DMEM containing FBS after 5 hours. In some cases, transfected HT-22 cells were treated with 100 nM DOPPA (Sigma) when transfection media was replaced with regular media. B12 cells were seeded (180,000 cells in a 60 mm cell culture dish) 24 hours prior to being treated with 100 nM DOPPA. For Aβ experiments, HT-22 and B12 cells were treated with 20 µM Aβ_1–42_ (California Peptide) for 24 hours. Cells were harvested after 24 hours of either DOPPA or Aβ treatment for immunoblot analysis.

Primary cortical neuronal cultures were derived from embryonic day 15 C57/BL6 mice. All animal procedures were performed in compliance the Canadian Council on Animal Care guidelines under an animal protocol (#2011-079) approved by Western University’s animal care committee. Cortices from each embryo were treated with trypsin (Sigma) to dissociate neurons, as described previously^102^. Neurons were then plated in 96-well plates (40,000 cells/well) pre-treated with poly-L-ornithine (Sigma) and cultured in neurobasal medium (Invitrogen) supplemented with 2 mM Glutamine (Life Tech), 50 units/ml penicillin and streptomycin (Invitrogen), and B27 (Invitrogen) and N2 (Invitrogen) supplements. Culture medium was replaced every three days. Cytosine Arabinoside (Sigma) was added to the culture medium on day 3 at a concentration of 5 µM to prevent any contaminating glial cells from propagating. Following 4 days in culture, neurons were treated with DOPPA and on day 6, neuronal cultures were treated with 20 µM of Aβ_1–42_ for 24 hours.

### Expression plasmids and siRNAs

A human p66SHC expression plasmid, generously provided by Dr. Mauro Cozzolino (Fondazione Santa Lucia IRCSS, Italy), was used as a template to generate an HA-tagged p66SHC cDNA by PCR, with forward primer sequence 5′-GACGATAGTCCGACTACCCTGTGT-3′ and reverse primer sequence 5′-ACTCTAGATTAAGCGTAGTCTGGGACGTCGTATGGGTACAGTTTC-CGCTCCAC-3′. Once amplified, the HA-tagged p66SHC cDNA was digested using EcoRI and XbaI restriction enzymes (ThermoFisher Scientific), and the digested product was then ligated into a pcDNA3.1 vector. Incorporation of the PCR product was then confirmed by sequencing. p66SHC specific and control siRNAs were purchased from ThermoFisher Scientific (siRNA IDs: p66Shc-1: 151656, p66Shc-2: 253836, and control- AM4611). Sequence for p66Shc-1 siRNA is 5′-GCUUUGUCAAUAAGCCCACTT-3′ (forward) and 5′-GUGGGCUUAUUGACAAAGC-TC-3′ (reverse), and the sequence for p66Shc-2 siRNA is 5′-UCCCAACGACAAAGUCAUGTT-3′ (forward) and 5′-CAUGACUUUGUCGUUGGGATG-3′ (reverse). For optimal *p66SHC* knockdown, both p66Shc-1 and p66Shc-2 siRNAs were combined in a 1:1 ratio during transfection. siRNA knockdown experiments were performed using the Lipofectamine RNAiMAX (ThermoFisher Scientific), according to manufacturer’s instructions. In brief, B12 cells were seeded in a 6-well cell culture plate 24 hours before siRNA transfection. The following day, p66Shc specific and control siRNAs were added to Opti-MEM (Gibco) to obtain a final siRNA concentration of 75 pmol, and then mixed with Opti-MEM containing Lipofectamine RNAiMAX, and incubated for 5 mins at room temperature. The siRNA-lipid complex in Opti-MEM was then added to the DMEM in each corresponding well in the cell culture plate and incubated at 37 °C and 5% CO_2_ for 36 hours. Cells were harvested 36 hours post transfection for immunoblot analysis.

### Immunoblot Analysis

Cells were washed twice in PBS and lysed in ice-cold RIPA buffer (10 mM Tris-Hcl pH 8, 1% Triton X-100, 0.1% Sodium deoxycholate, 0.5 mM EGTA, 0.1% SDS, 140 mM NaCl) containing a protease inhibitor cocktail (2 mM leupeptin (Sigma), 0.1 mM pepstatin A (Sigma)), phenolmethanesulfonyl fluoride (Sigma), and sodium orthovanadate (Sigma). The cell debris was removed by centrifugation at 16,000 g at 4 °C for 10 min and the resulting supernatant was collected. Protein concentrations were determined using the DC protein assay (Bio-Rad), and extracts were resolved by 10% SDS-PAGE. Separated proteins were immunoblotted onto polyvinylidene fluoride membrane (Bio-Rad), and blocked in TBS buffer containing 3% BSA (VWR) and 1% nonfat dry milk (Cell Signalling). The following primary antibodies were used: p66SHC (AM00143PU-N; Acris Antibodies), pSer^35^ p66SHC (566807; EMD Millipore), SHC (610878; BD Biosciences), HA-tag (MMS-101P; Covance), PDH (ab110334; abcam), Actin (sc-47778; Santa Cruz), pser^232^ PDH (AP1063; EMD Millipore), LDHA (#2012; Cell Signalling), PDK1 (ADI-KAP-PK112-F; Enzo Life Sciences), and PKM2 (#3198; Cell Signalling). HRP-conjugated secondary mouse (sc-2005; Santa Cruz) and rabbit (sc-2006; Santa Cruz) antibodies. Bands were detected using Luminata Forte chemiluminescence substrate (EMD Millipore) and immunoblots were imaged using a Chemidoc XRS System (Bio-Rad). Band density quantification was performed using Image Lab software (Bio-Rad).

### Fluorescence Microscopy

For visualizing mitochondrial membrane potential, the fluorescent dye TMRM (ThermoFisher Scientific) was used. Mitochondrial ROS production was measured using the fluorescent dye MitoTracker Red CMXRos (Life Technologies). HT-22 cells were seeded in 35 mm cell culture dishes, transfected as described earlier, and treated with DOPPA for 24 hours. B12 cells were seeded in 35 mm cell culture dishes overnight and treated with DOPPA for 24 hours. B12 cells transfected with p66Shc siRNA were trypsinized and seeded in 35 mm dishes cell culture dishes overnight before microscopic analysis. Cell culture medium was aspirated and replaced with phenol-red free DMEM (10% FBS, 1% pen/strep) containing either 200 nM TMRM or 200 nM MitoTracker Red CMXRos, and culture dishes were incubated at 37 °C and 5% CO_2_ for 20 minutes. Cells were then rinsed twice with PBS and incubated with PBS containing 10 µg/mL Hoechst (Life Technologies) for 1 minute at room temperature. Cells were further rinsed with PBS and imaged in phenol-red free DMEM (10% FBS, 1% pen/strep) using a Zeiss Axio Observer AI microscope. All images were captured at the same exposure time and were analyzed using ImageJ software (National Institute of Health).

### Cell Viability Assay

Viability of HT-22 and B12 cells, and primary cortical neurons was measured using the MTT assay. HT-22 cells were transfected in 60 mm dishes and treated with DOPPA for 24 hours as described above. Following DOPPA treatment, transfected HT-22 cells were trypsinized and seeded in a 96-well plate (7000 cells/well) in DMEM supplemented with 5% FBS and 1% pen/strep. After 5 hours of seeding, the culture medium was replaced with DMEM (5% FBS and 1% pen/strep) containing either 100 nM DOPPA or 20 µM Aβ_1–42_ for a period of 24 hours. B12 cells were seeded in a 96-well plate (10,000 cells/well) and 24 hours after DOPPA treatment, culture medium was aspirated and replaced with DMEM (5% FBS and 1% pen/strep) containing either 100 nM DOPPA or 20 µM Aβ_1–42_ for 24 hours. B12 cells transfected with p66Shc siRNA were trypsinized and seeded in a 96-well plate overnight, and cell culture medium was removed the following morning and replaced with DMEM (5% FBS and 1% pen/strep) containing 20 µM Aβ_1–42_ for 24 hours. Mouse primary cortical neurons were seeded in a 96 well plate as described above. Culture medium was changed every 3 days and treated with 100 nM DOPPA on the fourth day for 24 hours. At 5 days *in vitro* (DIV) and after 24 hours DOPPA treatment, culture medium was aspirated and replaced with neurobasal medium containing containing either 100 nM DOPPA or 20 µM Aβ_1–42_ for 24 hours. Following DOPPA and/or Aβ_1–42_ treatment, culture medium was replaced with DMEM (1% FBS and 1% pen/strep) and 3-(4,5-Dimethylthiazol-2-yl)-2,5-diphenyltetrazolium bromide (MTT; Sigma) was added at a final concentration of 10%. Culture plates were incubated at 37 °C and 5% CO_2_ for 3 hours. After incubation, culture medium containing MTT was replaced with DMSO and the optical density was measured at 595 nm using a microplate reader (Bio-Rad Model 3550). All treatments were seeded in triplicates.

### Seahorse XFe24 Mitochondrial Flux Analysis

B12 cells were plated at a density of 40,000 cells per well in DMEM (5% FBS and 1% pen/strep) in a Seahorse XFe24 cell culture microplate and incubated overnight in the absence or presence of 100 nM DOPPA. All wells were then washed twice with bicarbonate-free Seahorse XF assay medium (Agilent) supplemented with 10 mM glucose, 4 mM L-glutamine, and 1 mM sodium pyruvate, and pH adjusted to 7.35 +/− 0.05. Following washes, the cell culture plate was incubated in the XF assay medium for 1 hour at 37 ^o^C. Mitochondrial oxygen consumption rate (OCR) was first measured at baseline, and then sequentially after the administration of 1 µM oligomycin (Agilent), 1 µM FCCP (Agilent), and 0.5 µM rotenone/antimycin A (Agilent). After the Seahorse XF MitoStress Test assay, cells in control and treatment wells were lysed and protein harvested using RIPA buffer as stated earlier. Data was normalized by protein concentration.

### Statistical Analyses

All data presented here are expressed as the mean ± SEM of at least 3 independent experiments. Effects of the treatments were assessed using either a two-tailed Student’s *t*-test or ANOVA, using GraphPad Prism (version 6). The difference between mean values was considered statistically significant at p < 0.05.

## Electronic supplementary material

Supplementary Information
