# Supplementary material for: p66Shc activation promotes increased oxidative phosphorylation and renders CNS cells more vulnerable to amyloid beta toxicity
Source: Sci Rep. 2018 Nov 20;8:17081. doi: 10.1038/s41598-018-35114-y (PMC6244282; doi:10.1038/s41598-018-35114-y)

Supplementary Information

p66Shc activation promotes increased oxidative phosphorylation and renders CNS cells more vulnerable to amyloid beta toxicity

Authors: Asad Lone, Richard A. Harris, Olivia Singh, Dean H. Betts and Robert C. Cumming

Supplementary Figure Legends

Figure S1. Aβ exposure promotes p66Shc phosphorylation independent of JNK activation in B12 cells. (**A**) Immunoblot analysis of extracts from B12 cells treated with $A\beta_{1-42}$ (20μM) for 24 hours. p66Shc phosphorylation was significantly increased following Aβ treatment, whereas JNK phosphorylation was not affected. (**B**) Densitometric analysis of immunoblots. Data presented are the mean ± SEM of 3 independent experiments (*P<0.05, **P<0.01; ***P<0.001).

Figure S2. Aβ exposure promotes p66Shc phosphorylation independent of JNK activation in HT22 cells. (A) Immunoblot analysis of extracts from HT22 cells transfected with the p66Shc and pcDNA plasmids and treated with $A\beta_{1-42}$ (20 μM) for 24 hours. (**B**) Densitometric analysis of immunoblots revealed significantly higher p66Shc phosphorylation levels as a result of Aβ exposure. No change was observed in JNK phosphorylation levels following Aβ exposure. Data presented are the mean ± SEM of 3 independent experiments (*P<0.05, **P<0.01; ***P<0.001).

Lone et al. Figure S1

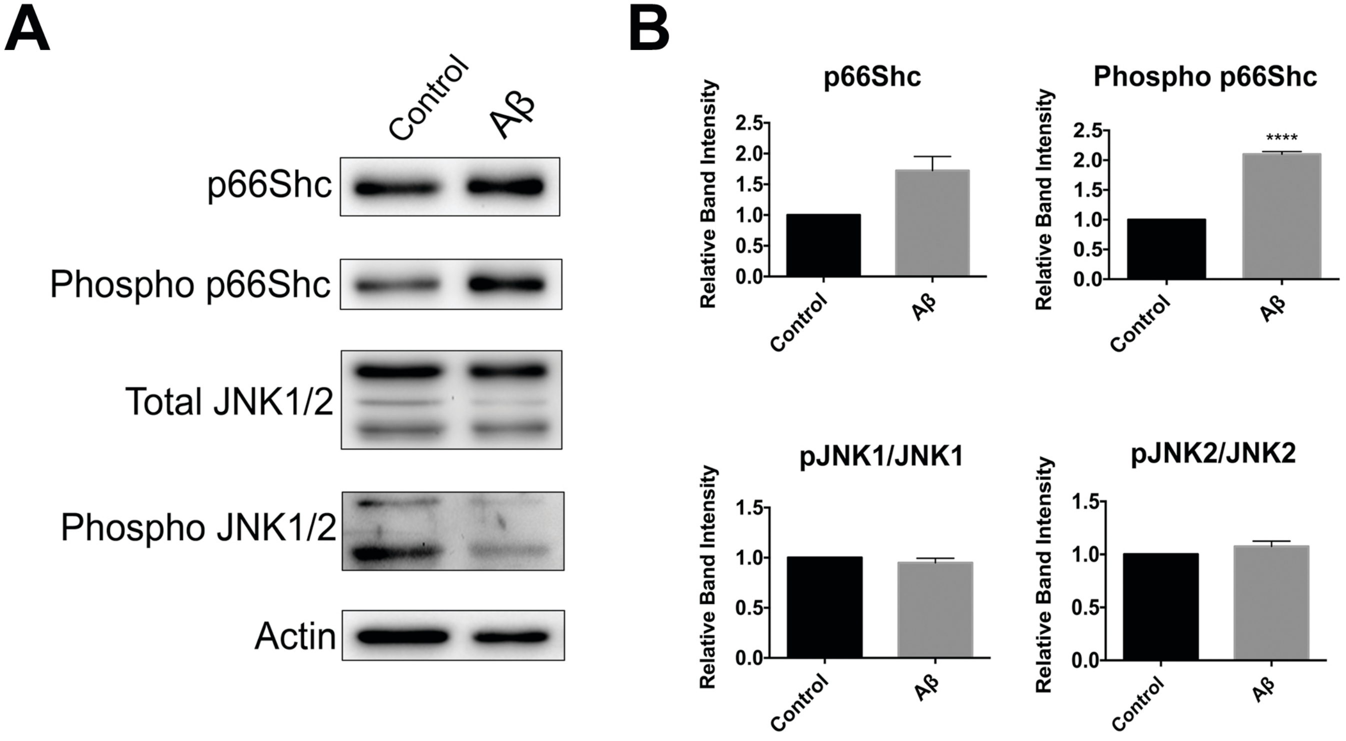

Lone et al. Figure S2

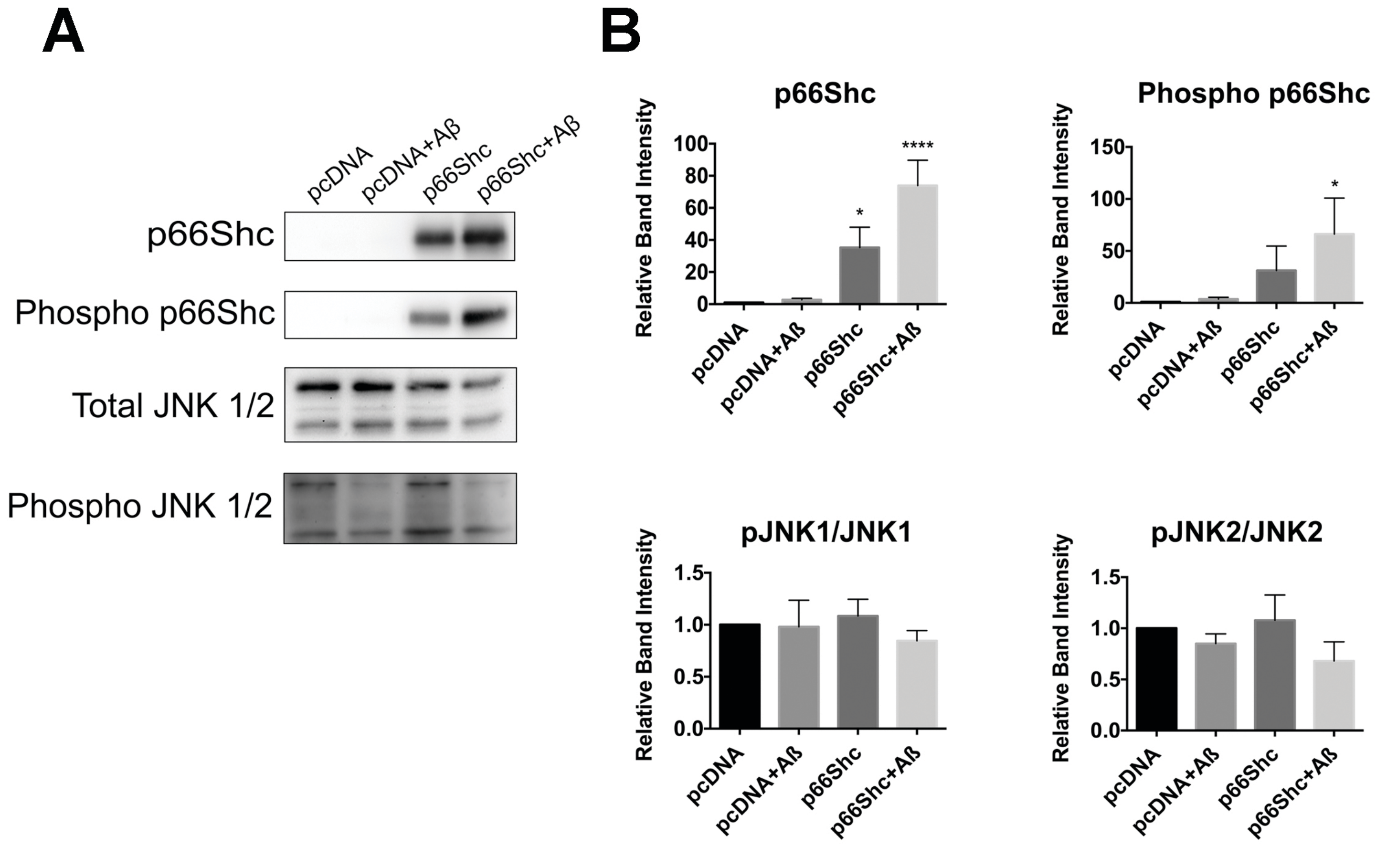

Supplement: Supplementary file 1 — Supplementary Information [file 41598_2018_35114_MOESM1_ESM.pdf]
